# Young Adults’ Short-Term Trajectories of Moderate Physical Activity: Relations With Self-Evaluation Processes

**DOI:** 10.3389/fpsyg.2020.02079

**Published:** 2020-09-11

**Authors:** Alex C. Garn, Kelly L. Simonton

**Affiliations:** ^1^School of Kinesiology, Louisiana State University, Baton Rouge, LA, United States; ^2^School of Health Studies, University of Memphis, Memphis, TN, United States

**Keywords:** emotions, exercise, motivation, self-efficacy, shame

## Abstract

Young adults face numerous barriers that can undermine their engagement in healthy behaviors. For example, young adults on average experience disproportionally large declines in physical activity (PA) participation compared to other demographic groups. Self-evaluation processes may help explain these declines. This study investigated young adults’ weekly trajectories of moderate physical activity, exploring self-evaluation processes, including self-efficacy and shame as time-varying covariates. A total of 71 young adults (*M*age = 21.25, *SD* = 1.18; 55% male) reported moderate physical activity, exercise self-efficacy, and anticipated shame toward exercise once a week for 5 weeks. Latent growth curve models showed that a linear slope fit these data better than alternative models. Parameters of the linear model revealed that these young adults reported engaging in 40 min of moderate PA approximately 3 days per week. However, there were physical activity differences in initial levels and rates of change. Exercise self-efficacy consistently predicted physical activity in a positive direction and with a small-to-medium magnitude. Anticipated shame was an inconsistent predictor of physical activity, showing a negative direction and small magnitude at time one and on average across the 5 weeks. These findings highlight considerable variability in young adults’ short-term trajectories of physical activity and underscore both positive and negative processes of exercise related self-evaluations. Future physical activity interventions targeting young adults should incorporate strategies that enhance self-efficacy (e.g., mastery experiences) and reduce feelings of shame (e.g., attribution training).

## Introduction

Regular participation in physical activity (PA) is a lifestyle habit that enhances physiological and psychosocial wellbeing across all segments of the population (United States Department of Health and Human Services, 2018). Regular PA also reduces risk for developing non-communicable diseases that can reduce quality of life and cause mortality (Piercy et al., 2018). In fact, evidence underscores increasing rates of preventable risk factors such as obesity and cardiovascular disease in younger populations (Wittekind et al., 2018). Young adults are one segment of the population who, on average, experience disproportionally large declines in regular PA during the transition from adolescence (Zick et al., 2007; Kwan et al., 2012). Among different segments of the young adult population, those who enroll in post-secondary education appear to be at greatest risk for experiencing declines in PA (Bray and Born, 2004). According to American College Health Association (2020), less than half of college and university students in the United States meet PA recommended guidelines related to aerobic activity and less than 40% related to muscular strength and endurance. In fact, evidence suggests that college and university students throughout the world often fail to meet PA guidelines (Haase et al., 2004).

PA participation is a complex endeavor; therefore, explaining declines in young adults’ PA is a difficult process. Combinations of environmental and biopsychosocial factors potentially cause variations in both short-term and long-term PA patterns. In general, many young adults who attend college or university experience major life changes such as moving away from home for the first time, having greater independence in day-to-day decision making, and experiencing changes in peer groups that for many appear to undermine health (Pullman et al., 2009; Deforche et al., 2015). Other factors such as work and/or school demands, sickness, competence beliefs, weather, access to facilities, and social support can facilitate or disrupt young adults’ PA on a daily, weekly, monthly, and/or yearly interval.

Although evidence suggests that many college and university students fail to meet PA guidelines (Haase et al., 2004), there remain gaps to understanding how young adults’ engagement in PA changes over time. For example, cross-sectional studies provide no information on changes in young adults’ PA behavior (e.g., Drenowatz et al., 2015). Intervention studies provide important information on how young adults’ PA behavior changes under controlled conditions but rarely explores intra-individual changes (e.g., Fiebert et al., 2004). Furthermore, these studies often assume homogeneity in PA behavior within the sample at baseline. There is a clear need for investigating within person trajectories of young adults’ PA, including naturally occurring factors that may help explain individual patterns (Lemoyne et al., 2016). Currently there is conflicting evidence on the nature of change in young adults’ PA with research demonstrating both increases (Lemoyne et al., 2016) and decreases (Lounassalo et al., 2019) over time. In this study, we investigate young adults’ weekly PA trajectories, examining whether self-evaluation processes help explain individual differences in these trajectories. Specifically, we explore two self-evaluation processes (Tracy and Robins, 2004; Baldwin et al., 2006): exercise self-efficacy and anticipated shame. In the paragraphs below, we define exercise self-efficacy and anticipated shame within the context of self-evaluation; describe how self-evaluation links to health behaviors such as PA; and outline the purpose and specific hypotheses of the study.

### Self-Evaluation and Physical Activity

People strive to maintain a positive sense of self, seeking out opportunities that facilitate positive and avoiding situations that produce negative self-evaluations (Baumeister, 1999; Marsh, 1990). Theorists suggest self-evaluation processes such as self-efficacy and shame influence how individuals interpret, act, and react within their environment (Bandura, 2004; Baldwin et al., 2006). Self-evaluation provides a common link between self-efficacy and shame. Bandura (1997) defines self-efficacy as one’s beliefs about capabilities to engage in behaviors that lead to specific performance achievements. Individuals rely on self-evaluation (e.g., “do I have the capability to run a mile in 8:00 min?”) when making judgments about their self-efficacy (Bandura, 2004). Shame also requires self-evaluation, representing a self-conscious, negative emotion whereby individuals believe they have or will fail to meet an internal and/or external standard, resulting in self-degradation (Tracy and Robins, 2004). Self-efficacy is central to health behaviors because people have little incentive to take action and persist unless they believe in their abilities to produce desired outcomes (Bandura, 2004). Likewise, feelings of shame can reduce health behaviors through ambivalence, aversion, and withdraw (Danielson et al., 2016). Together, self-efficacy (i.e., can I do it?) and shame (i.e., how will I feel if I fail to do it?) reflect common cognitive and emotional self-evaluation processes that direct behavioral engagement and previous research establishes connections between self-evaluation and various health behaviors, including PA (Castonguay et al., 2017).

Self-efficacy is arguably the single strongest self-evaluation determinant of PA adoption, adherence, and maintenance (McAuley and Blissmer, 2000; Wallace et al., 2000; Amireault et al., 2012). This evidence is robust across different demographics, time intervals, parameters of PA, study designs, and study contexts. For example, PA studies demonstrate that self-efficacy consistently enhances future PA in youth (Dishman et al., 2004), young adults (Parschau et al., 2012; Farren et al., 2017), adults (Dallow and Anderson, 2003), and older adults (Clark, 1996). However, Anderson-Bill et al. (2011) revealed that exercise self-efficacy tends to decline with age. Research also demonstrates that self-efficacy predicts changes in both short-term (Courneya and McAuley, 1994) and long-term (Amireault et al., 2012) PA. Courneya and McAuley (1994) also reported self-efficacy as a predictor of both frequency and intensity of PA in young adults.

Both observational (e.g., Farren et al., 2017) and intervention studies (e.g., McAuley et al., 2012) consistently highlight the importance of self-efficacy in relation to PA. For example, in a review of PA interventions (*n* = 20), Williams and French (2011) reported that the correlation between changes in self-efficacy and changes in PA was robust (*r* = 0.69). Self-efficacy is also a crucial determinant of PA maintenance. In a meta-analysis study of PA intervention studies conducted by Amireault et al. (2012), participants with higher levels of self-efficacy at baseline were more likely to be physically active and less likely to relapse 6 or more months post intervention. Finally, using self-efficacy as a tool for promoting PA can be across a variety of contexts, including school (Dishman et al., 2004), faith (Anderson-Bill et al., 2011), health (Maddison et al., 2014), and leisure (Farren et al., 2017) settings.

Self-efficacy is also theorized to affect other psychosocial factors of health behaviors such as goal setting and intentions (Bandura, 2004; Buchan et al., 2012) as well as affective responses (Magnan et al., 2013). Collectively, these robust links between self-efficacy and PA make it a central factor in the investigation of health behaviors. However, a majority of these studies has focused on the ability of self-efficacy to explain inter-individual differences in PA (Buchan et al., 2012). In other words, most studies examine how self-efficacy relates to differences or the average magnitude of change between groups of people. Few studies explore how self-efficacy relates to intra-individual changes in PA. This is a different research question that can potentially help uncover how well self-efficacy explains PA changes and its heterogeneity within individuals. This type of approach provides a dynamic perspective of understanding behavior change processes (Reuter et al., 2010).

Another appealing characteristic of focusing on self-efficacy to understand health behavior such as PA is the clear theoretical underpinnings that outline its enhancement. These characteristics include primary and vicarious experiences, verbal persuasion, and affective/physiological states (Bandura, 1997). Previous experiences, especially those considered successful, are considered the most prominent source of self-efficacy according to Bandura. Vicarious experiences represent the potential inspiration one gets from the observations of other people’s behavioral engagement. Verbal persuasion is a source of self-efficacy stemming from receiving positive feedback from others. Finally, affective and physiological states are the feelings one associates with their behavior. For example, this might be the vigor or enjoyment one links to PA. Meta-analysis studies suggest that mastery experiences, vicarious experiences, and verbal persuasion are the strongest sources for promoting PA self-efficacy (Ashford et al., 2010; Williams and French, 2011). Williams and French (2011) also highlighted the strategy of planning when, where, and how to be physically active when trying to boost self-efficacy.

While self-efficacy is arguably the most prominent self-evaluation process related to enhancing PA (McAuley et al., 2012), less is known about links between shame and PA. However, research is starting to highlight the salience of shame in undermining PA (Sabiston et al., 2010; Gilchrist et al., 2017). Emotions such as shame create multidimensional response tendencies over a relatively short period of time (Fredrickson, 2001). Emotions stem from personally meaningful appraisals of an object. For example, an individual invited to attend a new gym who anticipates experiencing scrutiny because of poor fitness is likely to avoid the situation (e.g., make up an excuse not to go). In this case, potentially losing social status in the eyes of others by showing incompetence is the object that provokes anticipated shame (Tracy and Robins, 2004). Common response tendencies associated with shame are thought to be disruptive, including avoidance, withdrawal, passiveness, and self-loathing (Gilbert, 1997; Haidt, 2003). However, some evidence suggests that shame can promote approach-oriented interpersonal responses such as cooperation and pro-social behavior (De Hooge et al., 2011).

In terms of PA, Sabiston et al. (2010) revealed that body-related shame was negatively related to leisure-time PA (*r* = −0.23) in a sample of adult women from Canada. Findings also revealed that body-related shame related to undesirable patterns of PA motivation (i.e., high controlled and low autonomous). Gilchrist et al. (2017) explored anticipated shame as a predictor of the quality and quantity of marathon training in the final 5 weeks leading up to the race. Findings revealed that anticipated shame was not associated with future time or effort spent training for the marathon. However, these participants reported very low levels of anticipated shame. The data collection timing may of also affected these results as it is common for marathon runners to reduce training (i.e., taper) in the final weeks of race preparation.

These studies provide a foundation from which to move future research on shame and PA forward. For example, Sabiston et al. (2010) focused on relations between body-related shame and PA. Future research needs to further clarify how the object focus of shame relates to PA. It is plausible that mapping the object focus of shame directly to the behavior (i.e., shame toward PA rather than shame toward body) may produce stronger relations with PA. Future research also needs to move beyond cross-sectional research designs with both men and women. Gilchrist et al. (2017) addressed both of these issues, however, their focus was on a highly active sample of adults (i.e., marathon runners) training for a competitive race. Thus, there is a clear need to examine links between shame and PA in samples that reflect broader populations of society.

### The Present Study

The purpose of this study was to investigate how self-evaluation processes operationalized as anticipated shame and exercise self-efficacy relate to young adults’ weekly trajectories of moderate intensity PA behavior. The first research question (RQ1) examines the nature of change in PA over the course of 5 weeks, focusing on the initial amount of PA, trajectories of PA over time, and the relation between these two aspects of PA. RQ2 investigates the amount of inter-individual difference in both the amount of PA and its trajectory. RQ3 and RQ4 investigate the extent to which exercise self-efficacy and anticipated shame (i.e., time-varying covariates) relate to participants’ PA, respectively.

## Materials and Methods

### Participants and Procedures

The sample included young adults (*N* = 71; *M*age = 21.25, *SD* = 1.18) from a large university in the Southeastern United States. There were slightly more males (*n* = 39, 55%) than females (*n* = 32, 45%). Participants mainly reported their race/ethnicity as White, Caucasian (66%), Black, African American (21%), or Multi-Racial (4%) and were seniors (82%), juniors (10%), and sophomores (8%) in their academic rank. The researchers’ Institutional Review Board provided reviewed and approved the study protocol. The researchers described the study to potential participants during one class period in a large Kinesiology course. The participants received an email with a link to an online survey each week for 5 consecutive weeks. During the first part of the first survey, participants read a passage that descried the voluntary nature of the study and provided informed consent by clicking on a button that started the survey. Each link was active for 48 h and sent to participants on each Monday morning.

### Measures

Participants completed questions about basic demographics, including age, sex, race/ethnicity, and grade classification. Exercise self-efficacy was based on an item from the Exercise Self-Efficacy Scale (McAuley, 1993): “How confident are you that you can exercise at a moderate intensity three times per week for at least 40+ min for the next week?” The answer scale ranged from 0% (not at all confidence) to 100% (highly confidence) using 10% intervals. Shame (Gilchrist et al., 2017) was measured with the following item “How ashamed will you feel if you do not meet your exercise goal this week?” on a scale ranging from 1 (not at all) to 5 (extremely). Finally, participants reported the number of days in the previous week they exercised for at least 40 min at a moderate intensity. This question was based on the Global Physical Activity Questionnaire developed by the World Health Organization (2005).

### Data Analysis

Preliminary analyses included examination of missing data, variable distribution patterns, descriptive statistics, and correlation estimates. In order to evaluate our main research questions, we tested a series of latent growth curve models within the structural equation modeling framework using Mplus version 7.4 (Muthén and Muthén, 2017). All models used maximum likelihood estimation procedures. Missing data were handled with full information likelihood estimation (FIML) procedures (Enders, 2010). Model fit was judged using chi-square estimates based on degrees of freedom, comparative fit index (CFI), Tucker-Lewis Index (TLI), root mean square error of approximation (RMSEA), and standardized root mean residuals (SRMR). For CFI and TLI, higher scores reflect better model to data fit with criteria of a good fit, ≥0.95, and acceptable fit, ≥0.90 (Hu and Bentler, 1999). For RMSEA and SRMR, lower scores represent better model to data fit with criteria of a good fit, ≤0.06, and acceptable fit, ≤0.08 (Hu and Bentler, 1999).

We started by testing unconditional latent curve models for moderate PA. Specifically, we tested a series of trajectories, including an intercept only model (i.e., no growth), linear trajectory, quadratic trajectory, and finally a latent basis trajectory. Time (i.e., slope) was coded as 0, 1, 2, 3, and 4 in the linear model. In the quadratic model, time was coded as 0, 1, 4, 9, and 16 in addition to the linear slope. Finally, in the latent basis model, time was coded 0 at T1 and 1 and T5, while T2–T4 were freely estimated (Ram and Grimm, 2007). Residual variance estimates were constrained to be equal across the five waves of data. We treated PA as a continuous variable because it had more than five categories and it was normally distributed (Sass et al., 2014; see Table 1).

**Table 1:** Descriptive statistics for all study variables.

	Total		Males		Females					
Variable	*M*	*SD*	*M*	*SD*	*M*	*SD*	Min	Max	Skew	Kurtosis
Week 1 PA	3.14	1.88	3.63	1.86	2.56	1.76	0	7	0.09	−0.43
Week 2 PA	3.36	1.80	3.76	1.67	2.94	1.86	0	7	−0.04	−0.41
Week 3 PA	3.23	1.59	3.58	1.60	2.87	1.52	0	7	0.06	−0.26
Week 4 PA	3.18	1.77	3.86	1.54	2.44	1.74	0	7	0.14	−0.52
Week 5 PA	3.32	1.65	3.94	1.56	3.62	1.50	0	7	−0.20	−0.26
Week 1 Shame	1.74	0.86	1.76	0.75	1.72	0.99	1	5	1.36	2.33
Week 2 Shame	1.80	0.95	1.76	0.89	1.84	1.01	1	5	1.19	1.15
Week 3 Shame	1.74	0.73	1.73	0.76	1.75	0.72	1	5	0.70	0.06
Week 4 Shame	1.84	0.99	1.77	1.00	1.91	0.99	1	5	1.10	0.07
Week 5 Shame	1.95	1.11	1.91	1.10	2.00	1.03	1	5	1.11	0.63
Week 1 ESE	7.61	2.58	8.34	2.00	6.75	2.94	0	10	−0.75	−0.80
Week 2 ESE	7.39	2.86	8.26	2.60	6.47	2.87	0	10	−0.59	−1.23
Week 3 ESE	7.40	2.77	8.42	2.21	6.34	2.92	0	10	−0.69	−0.80
Week 4 ESE	6.88	3.00	7.86	2.74	5.81	2.94	0	10	−0.42	−1.23
Week 5 ESE	7.10	2.72	8.12	2.34	5.93	2.70	0	10	−0.41	−1.16

PA, average number of days per week of 40+ min of moderate physical activity; ESE, exercise self-efficacy.

We then added both time-invariant and time-varying covariates. Specifically, sex (male = 1, female = 0) was added as a time-invariant predictor of PA, while anticipated feelings of shame and exercise self-efficacy were added as time-varying covariates. We ran two conditional models, the first whereby time-varying covariates were estimated at each time point, and the second whereby equality constraints were added to each time-varying covariate in order to obtain the standardized relationship for each covariate with the PA trajectory.

## Results

### Preliminary Findings

There were 322 time specific observations across the five waves of data. Approximately 82% of the participants completed all five waves of data, 12% four waves, 4% two waves, and 2% one wave. The amount of missing data at each time was: (a) week 1, 1%, (b) week 2, 7%, week 3, 8%, week 4, 5%, and week 5, 12%. Reports of PA produced normal distribution properties at each time point (see Table 1). Table 1 presents descriptive statistics for all study variables. These young adults reported engaging in 40 min of moderate PA approximately 3 days per week. Reports of shame were below the midpoint of its scale in all five waves of data whereas reports of exercise self-efficacy were above the midpoint of its scale in all five waves of data. A correlation matrix is provided in Table 2. In general, there were positive, moderate relations between PA and exercise self-efficacy at each time point. There were negative, weak-to-moderate relations between PA and shame at each time point.

**Table 2:** Correlation matrix for all study variables.

	ESE_T1	Shame_T1	PA_T1	ESE_T2	Shame_T1	PA_T2	ESE_T3	Shame_T3	PA_T3	ESE_T4	Shame_T4	PA_T4	ESE_T5	Shame_T5	PA_T5
ESE_T1	1														
Shame_T1	−0.377^**^	1													
PA_T1	0.670^**^	−0.459^**^	1												
ESE_T2	0.832^**^	−0.331^**^	0.530^**^	1											
Shame_T2	−0.245^*^	0.556^**^	−0.19	−0.226	1										
PA_T2	0.520^**^	−0.422^**^	0.612^**^	0.598^**^	−0.236	1									
ESE_T3	0.781^**^	−0.291^*^	0.608^**^	0.786^**^	−0.332^**^	0.448^**^	1								
Shame_T3	−0.370^**^	0.396^**^	−0.243	−0.323^**^	0.542^**^	−0.294^*^	−0.331^**^	1							
PA_T3	0.547^**^	−0.258^*^	0.677^**^	0.510^**^	−0.028	0.678^**^	0.507^**^	−0.215	1						
ESE_T4	0.739^**^	−0.279^*^	0.541^**^	0.702^**^	−0.237	0.401^**^	0.772^**^	−0.304^*^	0.403^**^	1					
Shame_T4	−0.346^**^	0.549^**^	−0.367^**^	−0.327^**^	0.485^**^	−0.264^*^	−0.316^*^	0.611^**^	−0.142	−0.321^**^	1				
PA_T4	0.526^**^	−0.243^*^	0.563^**^	0.424^**^	−0.166	0.530^**^	0.473^**^	−0.154	0.549^**^	0.578^**^	−0.249^*^	1			
ESE_T5	0.711^**^	−0.277^*^	0.653^**^	0.760^**^	−0.318^*^	0.479^**^	0.786^**^	−0.399^**^	0.514^**^	0.779^**^	−0.380^**^	0.573^**^	1		
Shame_T5	−0.296^*^	0.339^**^	−0.268^*^	−0.148	0.450^**^	−0.157	−0.176	0.582^**^	−0.148	−0.286^*^	0.688^**^	−0.317^*^	−0.298^*^	1	
PA_T5	0.433^**^	−0.127	0.542^**^	0.326^*^	−0.117	0.401^**^	0.421^**^	−0.155	0.485^**^	0.438^**^	−0.267^*^	0.752^**^	0.610^**^	−0.289^*^	1

PA, average number of days per week of 40+ min of moderate physical activity; ESE, exercise self-efficacy; T1, time 1.

* *p* < 0.05;

** *p* < 0.01.

### Main Findings

Table 3 provides results from unconditional latent growth curve model testing. Our findings revealed that the linear latent growth curve model for PA fit these data better than the intercept-only model (Δ*CFI* = 0.051), and fit these data equally well compared to the more complex quadratic and latent models. Therefore, we used the linear model for subsequent analyses. The overall fit was acceptable although the RMSEA estimate was slighter higher than recommended values (Hu and Bentler, 1999). Table 3 highlights parameter estimates of the linear latent growth curve model for PA. The latent intercept mean revealed that the predicted level of participants’ PA consisted of engaging in 40 min of moderate intensity PA on 3.187 days during week 1 with meaningful variance around the mean. The latent slope mean estimate was not statistically significant, suggesting that on average PA trajectories were stable across the 5-week period. However, the linear slope variance estimate underscored heterogeneity in the PA trajectories. The negative covariance between the intercept and linear slope implies that participants’ reporting more days of PA at week 1 experienced a slower rate of change compared to participants reporting fewer days of PA at week 1.

**Table 3:** Latent growth model fit statistics.

Model	*χ* ^2^	*df*	CFI	TLI	RMSEA	SRMR
Physical activity						
Intercept only	34.527^**^	17	0.897	0.939	0.121	0.114
Linear	22.882	14	0.948	0.963	0.095	0.079
Quadratic	20.237^*^	10	0.940	0.940	0.120	0.097
Latent	19.892^*^	11	0.948	0.952	0.107	0.102
Conditional free	73.520^*^	53	0.901	0.879	0.082	0.047
Conditional standardized	80.879^*^	61	0.904	0.898	0.075	0.065

Intercept only model represents a “no growth” model; linear, linear slope with time coded 0, 1, 2, 3, and 4. Quadratic model includes linear and quadratic slopes with quadratic slope coded 0, 1, 4, 9, and 16. Latent model coded 0 at T1 and 1 at T5 while T2–T4 are estimated. Bottom part of table reflects. Latent growth models with covariates. In the free model, time varying covariates are estimated at each time point. In standardized model, time varying covariates are constrained to be equal across five time points.

* *p* < 0.05:

** *p* < 0.01.

Model fit results from the conditional models, which included sex (male = 1, female = 0) as a time-invariant covariate, and shame and exercise self-efficacy as time-varying covariates are provided at the bottom of Table 3. We tested two conditional models; the free conditional model estimated shame and exercise self-efficacy freely at each of the five time points while the standardized model placed equality constraints on shame and exercise self-efficacy, respectively, in order to examine the systematic relations across time. Adding these equality constraints produced a similar fitting model compared to the freely estimated model. Therefore, we focus on the standardized model while addressing RQ3 and RQ4. The overall model fit was adequate, with only the TLI estimate (0.898) slightly below the recommended guideline of 0.90 (Hu and Bentler, 1999). Sex was not associated with participants’ predicted level of PA at week 1 or its rate of change (see Table 4). Support of the standardized model suggested that the time-varying covariates were stable across individual time points. A one-unit increase in shame was associated with a 0.225 decrease in PA during any given week whereas a one-unit increase in exercise self-efficacy was associated with a 0.279 increase in PA during any given week (see Table 4). The *R* ^2^ values for PA at each week in the final model were: (a) week 1, 0.676; (b) week 2, 0.616; (c) week 3, 0.597; (d) week 4, 0.683, and (e) week 5, 0.816. The *R* ^2^ values for latent intercept and slope were small, 0.037 and 0.010, respectively.

**Table 4:** Parameter estimates for linear latent growth models.

Parameter-unconditional	I mean	I variance	S mean	S variance	I S covariance	I S correlation
PA	3.187 (0.210)^**^	2.467 (0.527)^**^	0.025 (0.054)	0.088 (0.035)^*^	−0.204 (0.110)^*^	−0.533^*^
Parameter-conditional						
Physical activity	1.018 (0.439)^**^	1.178 (0.338)^**^	0.035 (0.080)	0.104 (0.036)^**^	−0.214 (0.094)^*^	−0.631^*^
Covariates	B (SE)	β				
I on Male	0.424 (0.359)	0.192				
S on Male	0.061 (0.112)	0.094				
PA_1 on ESE_1	0.369 (0.063)^**^	0.510				
PA_1 on Shame_1	−0.503 (0.173)^*^	−0.241				
PA_2 on ESE _2	0.305 (0.053)^**^	0.491				
PA_2 on Shame_2	−0.229 (0.149)	−0.127				
PA_3 on ESE _3	0.261 (0.047)^**^	0.434				
PA_3 on Shame_3	−0.154 (0.164)	−0.068				
PA_4 on ESE _4	0.258 (0.047)^**^	0.454				
PA_4 on Shame_4	−0.183 (0.128)	−0.127				
PA_5 on ESE_5	0.229 (0.057)^**^	0.388				
PA_5 on Shame_5	−0.139 (0.132)	−0.111				
PA on ESE_ave	0.279 (0.040)^**^	0.413, 0.482				
PA on Shame_ave	−0.225 (0.093)^*^	−0.098, −0.146				

I, latent intercept; S, latent slope; PA, average number of days per week of 40+ min of moderate physical activity; ESE, exercise self-efficacy; B (SE), unstandardized beta coefficient with standard error; β, standardized beta coefficient. Ave, unstandardized beta average estimate across all five time points.

* *p* < 0.05;

** *p* < 0.01.

## Discussion

The purpose of this study was to investigate how self-evaluation processes relate to young adults’ weekly trajectories of moderate intensity PA behavior. Specifically, we examined four research questions related to PA trajectories (RQ1 and RQ2) and self-evaluation covariates (exercise self-efficacy, RQ3; anticipated shame, RQ4) over a 5-week period using latent growth modeling. Young adults, especially those who enroll in universities, are one segment of the population that typically experiences declines in PA (Kwan et al., 2012) and often fail to meet recommended levels of PA (Farren et al., 2017; American College Health Association, 2020). Major findings revealed considerable variation in young adults starting levels and trajectories of PA as well as consistent positive relations with exercise self-efficacy and inconsistent negative relations with anticipated shame.

### Weekly Trajectories of PA

RQ1 and RQ2 focused on young adults PA. Regular PA is an essential strategy for maximizing health and reducing modifiable risks causing morbidity and mortality (United States Department of Health and Human Services, 2018). Young adults attending university are especially vulnerable for experiencing declines in PA (Maselli et al., 2018). Findings from this study illustrate PA patterns in a sample of young adults from a region in the United States with consistently higher rates of sedentary behavior and obesity (Centers for Disease Control and Prevention, 2020). According to the United States Department of Health and Human Services (2018) adults should be active most days per week. On average, the participants in this study reported being active approximately 3 days per week with trajectories that stayed stable across the 5 weeks of the study. However, there was heterogeneity in both the initial levels and weekly trajectories of these young adults PA. Furthermore, young adults who reported more days of PA at baseline were more likely to see slower rates of change across the 5-week period. Although not directly comparable, these participants reported slightly lower amounts of PA compared to previous studies measuring young adults PA using accelerometers (Henderson et al., 2020).

The heterogeneity in our participants’ initials levels and rates of change in PA yield insights that can guide promotion strategies of short-term PA in young adults. Specifically, the variation in initial levels and trajectories of PA suggest the need for deliberate target strategies for young adult subgroups. Interestingly, our findings revealed that it is not as simple as targeting males or females. Previously effective strategies for university students include tailoring interventions to current levels of PA (Keating et al., 2005). For example, strategies for young adults who are completely sedentary would be different from strategies for those who participate in some PA. Furthermore, exploring profiles of specific sets of salient barriers toward PA may also help create more fine-tuned PA targeting. For example, young adults who report low levels of social support may need different intervention strategies than those who lack safe, accessible PA infrastructure.

Finally, offering university courses that promote health education and provide young adults with structured PA opportunities on a weekly basis may also help reduce the variability seen in this study. Adherence to PA is influenced by numerous factors, however, structure and supervision are often highlighted as key elements (Gilli et al., 2018). Unfortunately, many universities in the United States do not require students to enroll in personal health courses. Recent estimates suggest that only about 10% of universities in the United States required a personal health course in order for students to graduate (Henry et al., 2017).

### Self-Evaluation Predictors

Self-efficacy and shame share foundations grounded in self-evaluation (Baldwin et al., 2006). While self-efficacy has been widely acknowledged as a key determinant of PA (Dishman et al., 2004; Maddison et al., 2014), less research has examined the role of shame in shaping one’s PA. Findings from our study provide greater insights concerning self-efficacy (RQ3), shame (RQ4), and young adults’ short-term PA trajectories. Exercise self-efficacy was a consistent, positive predictor of PA at each of the five time points and on average across time. These results reinforce the importance of addressing one’s self-efficacy when considering the promotion of health behavior (Bandura, 2004). Therefore, targeting sources of self-efficacy represents a key consideration when developing PA interventions with young adults (Bandura, 1997; Ashford et al., 2010; Williams and French, 2011). However, findings from meta-analysis studies on enhancing self-efficacy toward PA specifically highlight divergences from standard suggestions of mastery experiences, verbal persuasion, vicarious experiences, and physiological/affective states of Bandura (1997).

In terms of mastery experience, which Bandura (1997) highlights as the most critical source of self-efficacy, Ashford et al. (2010) found that intervention techniques that used feedback on past performance was an especially successful strategy for enhancing one’s exercise self-efficacy. Similarly, Williams and French (2011) reported that feedback focused on effort or PA progress to be an effective strategy for enhancing exercise self-efficacy. Thus, developing PA environments that focus on feedback geared toward personal improvement and success appears warranted. However, both of these meta-analysis studies revealed that grading performance to mastery criteria actually lowered one’s exercise self-efficacy.

Interestingly, in the Ashford et al. (2010) meta-analysis, PA interventions that used vicarious experiences generally increased self-efficacy while persuasion techniques were associated with decreases in exercise self-efficacy. Thus, there appear to be nuances regarding how sources of self-efficacy can be implemented effectively in PA interventions. One of the most interesting findings from the Williams and French (2011) meta-analysis was the potential strength of using action planning techniques in PA interventions. Action planning is a self-regulation strategy aimed at creating if-then plans that help link environmental cues to behavioral responses (Conner et al., 2010). These if-then plans typically focus on addressing specific details about how, when, and where PA will occur as well as identifying contingences if/when barriers arise. Williams and French (2011) reveal that the use of action plan strategies in PA interventions enhance exercise self-efficacy and increased levels of PA. Therefore, future research should explore action planning techniques in health-related interventions because of their potential to facilitate both cognitive determinants and behavior.

Shame produced an inconsistent pattern of relationships with PA compared to exercise self-efficacy. Specifically, it was a negative predictor of PA at T1 and on average across the 5 weeks. However, it was not a significant predictor at T2, T3, T4, or T5. Our results produced both similarities and differences compared to previous research. For example, on average, participants in this study reported low levels of shame, similar to previous research with adult women (Sabiston et al., 2010) and runners training for a marathon (Gilchrist et al., 2017). The negative direction and low magnitude of the relationship between shame and PA was similar to findings from Sabiston et al. (2010). However, the average relations between shame and PA across time were a unique finding, diverging from results reported by Gilchrist et al. (2017). This is likely a reflection of differences in sample characteristics (i.e., somewhat active young adults versus adults in the final stages of marathon training).

Theorists suggest emotions produce predictable action tendencies over short periods of time (Fredrickson, 2001) with shame facilitating avoidance behaviors (Haidt, 2003). Unlike broader measures of affect, discrete emotions such as shame always have a specific object focus (Pekrun, 2006). In this study, the object focus of shame was not meeting one’s exercise goal for the week. The personal nature of an exercise goal may help explain the low magnitude in relation between shame and PA. Future researchers should consider creating a normative object focus of PA related shame such as failing to meet important others’ expectations or losing social status. It is possible that the normative implications of PA related shame may increase the magnitude of avoidance tendencies. In many instances, PA environments can facilitate social comparisons that lead to negative thoughts and feelings in young adult populations (Fitsimmons-Craft et al., 2016). In simple terms, using a normative object focus may increase the stakes related to one’s anticipated negative outcomes.

It is important to acknowledge limitations associated with this study. First, because of the repetitive nature conducting weekly data collections, we used one-item measures of all study variables to reduce participant burden. Despite robust auto-correlations across time (i.e., test-retest) for all variable (see Table 2), this is not an ideal measurement approach. Future researchers should use more comprehensive measures of exercise self-efficacy, shame, and PA. Second, we examined young adults’ weekly trajectories of PA. This short-term timeline reflects the theorized properties of emotional effects (Fredrickson, 2001) and follows previous studies on shame (Gilchrist et al., 2017), but may not generalize to long-term PA. Examination of longer measurement intervals is needed in future research. Third, we used a small sample of young adults from one university in one region of the United States. Future studies would benefit from examining self-evaluation and PA in diverse samples. Finally, we relied on self-report measures of PA, which may produce social desirability bias. Future research should use objective measures of PA such as accelerometers.

## Conclusion

The purpose of this study was to examine young adults’ weekly trajectories of PA, exploring exercise self-efficacy and anticipated shame as time varying predictors. Our findings demonstrated considerable variability in these young adults reports of PA at the beginning of the study and trajectories across time. Results also highlighted the positive and negative sides of PA self-evaluations. Self-efficacy continues to be one of the most consistent enhancers of PA, making it a strategic factor when considering future health-related interventions with young adults. Feelings of shame represented a barrier to these young adults’ PA, although its impact did not appear overly deleterious. Nevertheless, addressing situational factors that cause PA related shame needs greater examination in order to maximize young adults’ participation in health-enhancing PA.

## Data Availability Statement

The raw data supporting the conclusions of this article will be made available by the authors, without undue reservation.

## Ethics Statement

The studies involving human participants were reviewed and approved by Louisiana State University Institutional Review Board. The patients/participants provided their written informed consent to participate in this study.

## Author Contributions

AG helped conceptualize the study, analyzed the data, and contributed to the writing of the manuscript. KS helped conceptualize the study, collected the data, and contributed to the writing of the manuscript. All authors contributed to the article and approved the submitted version.

### Conflict of Interest

The authors declare that the research was conducted in the absence of any commercial or financial relationships that could be construed as a potential conflict of interest.
